# Dental Instrumentation Leading to Multivalvular Vegetation Endocarditis in an Otherwise Healthy Immunocompetent Patient Requiring Double Valve Replacement

**DOI:** 10.7759/cureus.78011

**Published:** 2025-01-26

**Authors:** Arnold Steinlage, Andrew J Evans, Christopher M Russo, Lydia Luu, Patrick Coleman

**Affiliations:** 1 Anesthesiology, Walter Reed National Military Medical Center, Bethesda, USA; 2 Medicine, Uniformed Services University of the Health Sciences, Bethesda, USA; 3 Anesthesiology and Critical Care, Walter Reed National Military Medical Center, Bethesda, USA

**Keywords:** adult cardiac surgery, dental infection, surgical replacement of valve, transesophageal echocardiography (tee), valvular endocarditis

## Abstract

Infective endocarditis (IE) is a severe condition associated with significant morbidity and mortality, often caused by bacterial seeding. Dental procedures are a well-known and well-documented risk factor for this disease. The authors present a case involving an otherwise healthy 58-year-old male with no cardiac risk factors who developed IE following a dental crown preparation. The patient’s dental-induced IE could not be managed medically due to bacterial abscess formation in the mitral and aortic valves. As a result, he developed both mitral and aortic valvopathies, characterized by mild mitral and severe aortic regurgitation, ultimately necessitating mitral valve and aortic valve replacement under cardiopulmonary bypass. This case report highlights the appropriate identification of IE, perioperative evaluation, intraoperative anesthetic management, and a review of echocardiographic findings. A heightened level of clinical awareness was critical for identifying this high-morbidity and high-mortality disease process. The report also reviews the full spectrum of diagnostic and therapeutic interventions in managing an otherwise healthy individual.

## Introduction

Infective endocarditis (IE) is an infection involving the endocardial tissues, commonly affecting the valves, mural endocardium, and implanted indwelling devices [1]. While IE is uncommon, with an estimated incidence of approximately 13.8 per 100,000 people, it carries a high in-hospital mortality rate of 15% to 20% and a one-year mortality rate of nearly 40% [1-3]. Dental procedures are among the most commonly performed worldwide and are well-documented as a risk factor for developing IE in both immunocompetent and immunocompromised patients. This association arises from the significant bacterial load and species variation in the oral cavity and gingiva. Instrumentation of this highly vascularized tissue can result in transient bacteremia, often asymptomatic and clinically silent in most dental patients. However, a small percentage of patients with known susceptibilities or medical risk factors can develop IE, which is challenging to diagnose and treat effectively [4]. Early diagnosis and immediate antibiotic therapy are essential, but nearly half of these cases also require cardiac surgery due to persistent infection, heart failure, or other complications [5]. Here, we report a case of an otherwise healthy 58-year-old male without major risk factors who, during a crown preparation procedure, developed dental-induced IE that could not be managed medically due to bacterial abscess formation on the mitral and aortic valves, necessitating a double valve replacement.

## Case presentation

A 58-year-old male with no past medical history presented to the emergency department (ED) with several days of progressive fever, chills, and fatigue approximately one month after an uncomplicated dental procedure (crown preparation and placement). Approximately 10 days after the dental procedure, the patient reported beginning to “feel ill.” After a clinic appointment with his family physician the following week, he was prescribed a 14-day course of doxycycline for presumed bacterial sinusitis. Despite the initiation of antibiotics, the patient’s symptoms continued to worsen, and his family physician instructed him to go to the nearest ED.

On presentation to the ED, the patient was hemodynamically stable, alert, and oriented. His initial vital signs included a temperature of 38.5°C, a heart rate of 119 beats per minute, a respiratory rate of 14 breaths per minute, a blood pressure of 134/65, and an oxygen saturation of 97% on room air. Auscultation of the precordium revealed a grade 3/6 systolic murmur, while breath sounds were clear bilaterally. Diagnostic tests were performed, including a complete blood count, a comprehensive metabolic panel, coagulation studies, blood cultures, urinalysis, and a CT scan of the abdomen and pelvis, and were all within normal limits.

Due to the patient’s history of recent dental instrumentation in the context of persistent fevers, he was admitted to the hospital, and an infectious disease specialist was consulted. The infectious disease team recommended obtaining repeat blood cultures and performing a transthoracic echocardiogram (TTE). A TTE was performed a few hours after admission and revealed a new diagnosis of acute severe aortic regurgitation with an ejection fraction of 60-65%.

**Figure 1:**
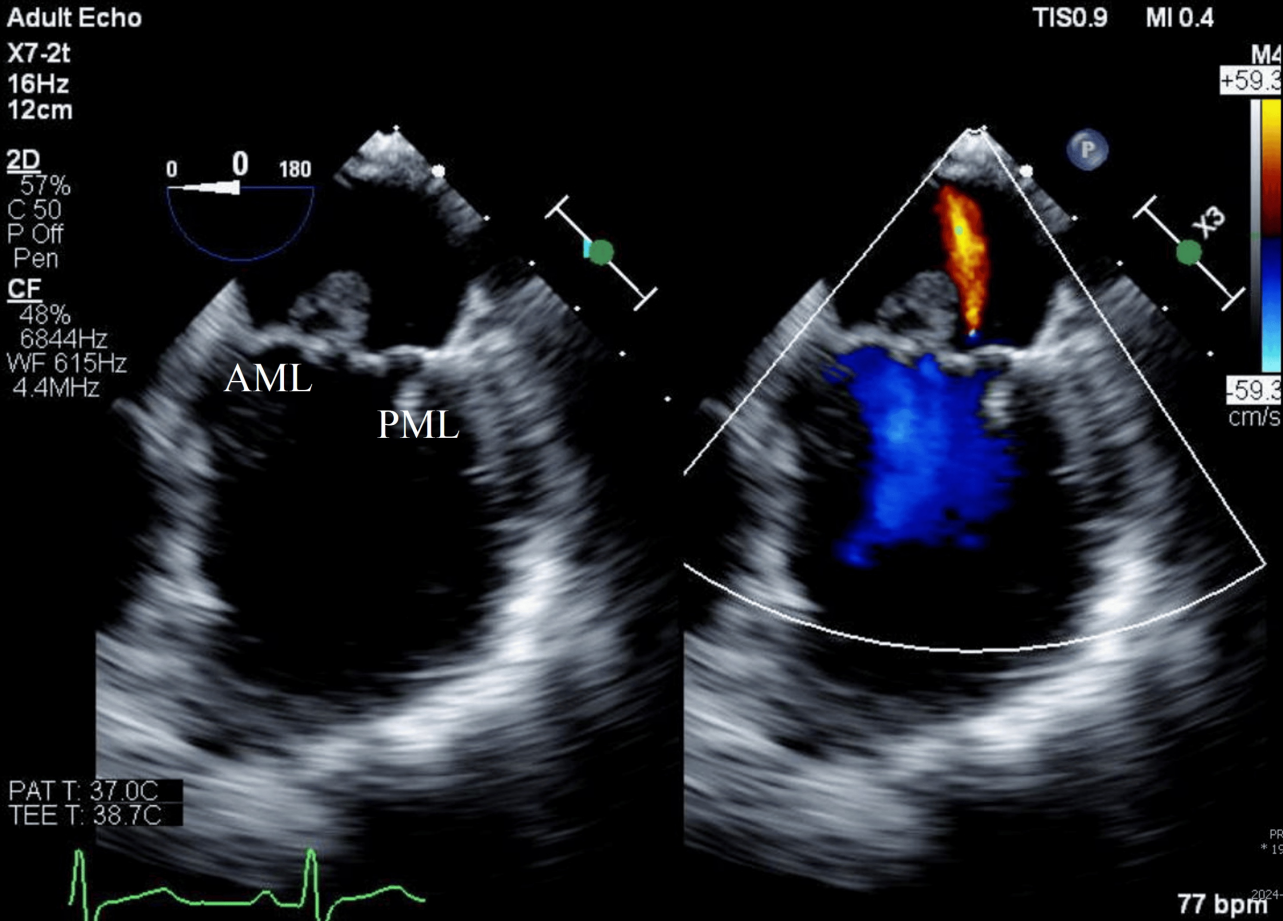
TEE mid-esophageal four-chamber view, mitral valve-focused showing large vegetation on the mitral valve with color Doppler showing mitral regurgitation TEE: transesophageal echocardiography, AML: anterior mitral leaflet, PML: posterior mitral leaflet

**Figure 2:**
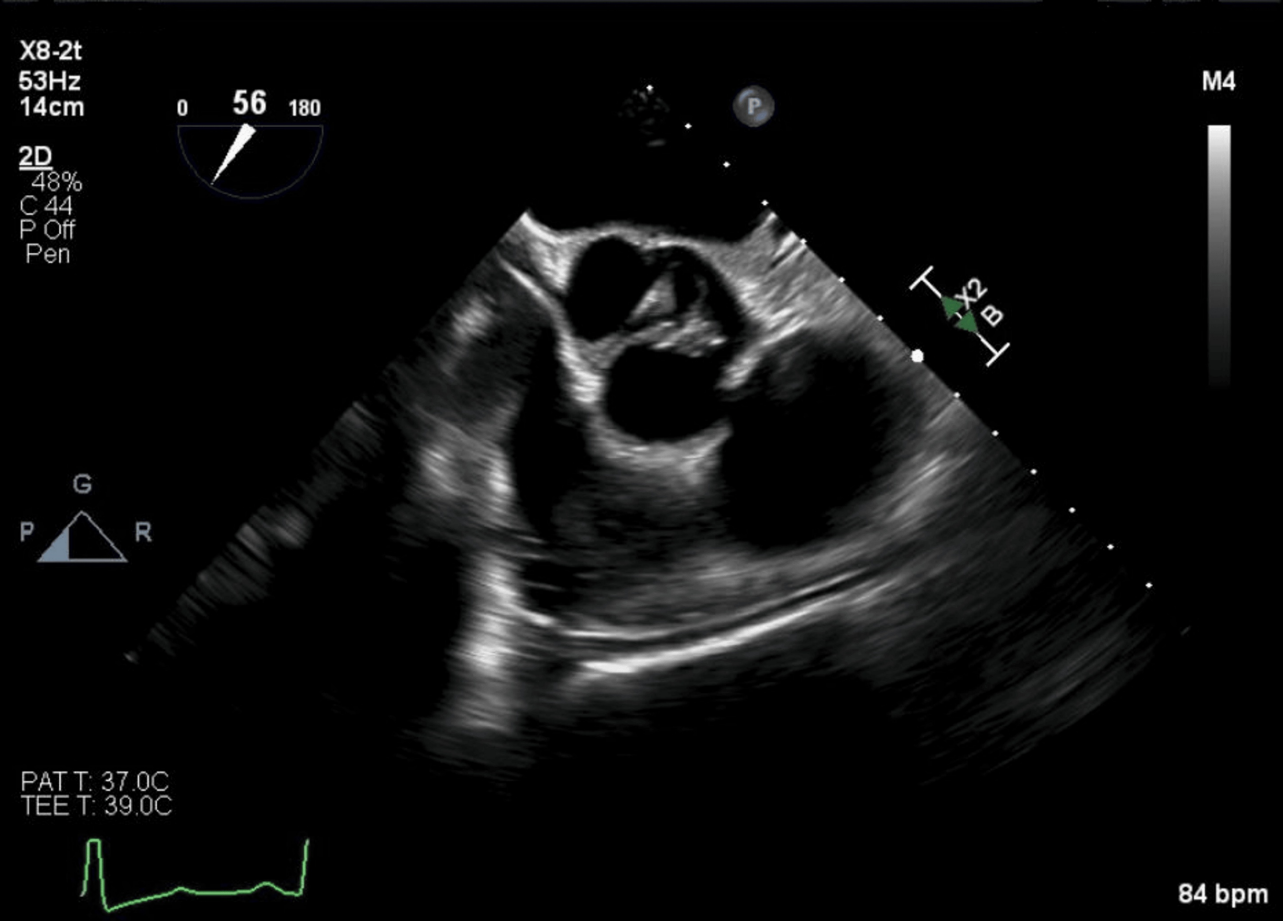
TEE aortic valve short axis view showing vegetation present at the commissure of the left and right coronary cusps TEE: transesophageal echocardiography

Given the positive findings suggestive of possible endocarditis on TTE and to further evaluate valvular involvement, cardiology performed a transesophageal echocardiogram (TEE) the following day. The TEE showed a large echogenic structure attached to the atrial side of the anterior mitral leaflet, measuring 1.4 × 1.1 cm and causing severe regurgitation (Figure 1). A similarly sized echogenic structure was noted on the aortic valve, with severe aortic regurgitation secondary to left coronary cusp rupture caused by vegetation (Figure 2).

Blood cultures were positive for *Streptococcus agalactiae*, and the patient was started on ceftriaxone for antibiotic coverage. Following consultation with a cardiothoracic surgeon, surgical intervention, including likely aortic and mitral valve replacement, was recommended if the patient’s symptoms persisted upon completion of his current antibiotic course.

The patient was brought to the operating room six days after his initial admission and upon completion of his antibiotic course. A pre-induction right radial arterial line was placed, general anesthesia was induced with intravenous agents, and the patient was endotracheally intubated without issue. Volatile-based anesthesia was used to maintain general anesthesia. A right-sided internal jugular central venous catheter and a pulmonary artery catheter were placed. The initial pulmonary artery pressure, central venous pressure, cardiac output, and cardiac index were within normal limits. Median sternotomy was performed, and the heart was suspended in the pericardial well. At this time, the patient remained hemodynamically stable and required no vasoactive medication support.

Following cannulation of the ascending aorta, the superior vena cava and inferior vena cava were cannulated for venous drainage. The patient was heparinized with 30,000 units of heparin, and after three minutes, the activated clotting time was 480 seconds. Cardiopulmonary bypass (CPB) was initiated. The native mitral valve and vegetation were resected, and a 29 mm Mitris Resilia valve was seated. After the mitral valve replacement, the aortic valve was resected, and a 27 mm Inspiris valve was placed. Intraoperative TEE demonstrated baseline biventricular function upon weaning from CPB, with no evidence of aortic or mitral regurgitation. There was no evidence of a prosthetic perivalvular leak, and each valve appeared to function normally with expected transvalvular gradients. The patient tolerated weaning from CPB and was decannulated.

The estimated blood loss was 650 milliliters, and no blood products were transfused intraoperatively. Chest tubes were placed, and the sternum was closed with wires in the standard fashion. The patient remained intubated, sedated, and hemodynamically stable upon arrival in the intensive care unit (ICU). The postoperative course was uncomplicated, and the patient was extubated six hours after arriving at the ICU without complications. A follow-up TTE on postoperative day five showed no perivalvular leaks, no evidence of malposition, no regional wall motion abnormalities, and an unchanged ejection fraction from baseline.

The patient experienced a rapid recovery, with resolution of symptoms, and could return to performing baseline daily activities by postoperative day seven. The patient was discharged with close follow-up from infectious disease specialists, who recommended a one-month course of antibiotics.

## Discussion

IE results from bacterial colonization of endocardial tissue, leading to vegetations composed of local platelet and fibrin deposits, which form in response to acute inflammatory reactions triggered by endothelial damage [5]. Regardless of the etiology, bacteremia, even if transient, is widely recognized as a prerequisite for the development of IE. The most common bacterial species implicated in IE are *Staphylococci*, *Enterococci*, and *Streptococci*, which have a strong affinity for adhering to tissues with platelet aggregation and fibrin deposition, resulting in vegetations. These bacterial vegetations initiate a cycle that perpetuates the inflammatory cascade, further promoting thrombus formation in a self-sustaining process [1,6].

Biofilms also develop, insulating bacteria from antibiotics; in some cases, surgical intervention is required to disrupt the biofilm and repair the damaged cardiac tissue. While the incidence of IE associated with invasive dental procedures remains unclear, both the American Heart Association (AHA) and the European Society of Cardiology (ESC) recommend restricting antibiotic prophylaxis for dental procedures to specific at-risk populations [3,7,8]. These populations include patients with a history of valvular surgery or replacement, organ transplant, or an immunocompromised state [8].

For a patient without the aforementioned risk factors and medical comorbidities to develop IE requiring multiple valve replacements is exceedingly rare, with only a few cases reported in the literature.

IE should be suspected in patients with known risk factors and specific symptoms, such as fever and malaise, and/or certain physical exam findings [3,5]. Cardiac risk factors for developing IE include valvular heart disease (both congenital and acquired), prosthetic valves, and a prior history of IE [3]. Non-cardiac risk factors include older age, male sex, central venous access, history of IV drug use, immunocompromised state, recent dental or surgical procedures, poor dental hygiene, and patients on hemodialysis [3].

On physical examination, most patients with valvular endocarditis present with fever (77.6%) and heart murmur (64.5%). Occasionally, symptoms of congestive heart failure, such as shortness of breath, exertional dyspnea, fatigue, and peripheral edema (27.2%), may also be observed. Non-specific symptoms, such as fever and malaise, can also occur [1].

The majority of IE cases are diagnosed within 30 days of symptom onset, so classic features such as petechiae, renal disease, and immunologic abnormalities are often absent. Classic exam findings, such as Janeway lesions (3.5%), Osler’s nodes (1.9%), and Roth’s spots (1.4%), though traditionally associated with IE, are exceedingly rare in clinical practice [1].

The evaluation and diagnosis of IE currently follow the 2023 Duke-International Society for Cardiovascular Infectious Diseases Criteria for IE (2023 Duke-ISCVID), including clinical and pathological criteria [9]. Positive blood cultures drawn before the initiation of antibiotics and echocardiographic evidence of IE serve as major diagnostic criteria for diagnosis [9]. Echocardiography is the initial imaging modality and the first diagnostic test performed when IE is clinically suspected and should be conducted promptly [5]. A TTE should be performed first due to its rapid and non-invasive nature. A TEE should be performed if the TTE results are positive or non-diagnostic in high suspicion for IE [10]. When TEE is contraindicated or unavailable, ECG-gated CT and/or fluorine-18 fluorodeoxyglucose PET/CT may be utilized [11].

Echocardiographic findings can include vegetations on a valve, other endocardial structures, or an implanted intracardiac device; valve or leaflet perforation or aneurysm; abscess; pseudoaneurysm; fistula; new significant valvular regurgitation; and new partial dehiscence of a prosthetic valve [9,12]. Compared to TTE, TEE has greater sensitivity and specificity for detecting valvular vegetations on both native and prosthetic valves, though sensitivity and specificity are slightly reduced with prosthetic valves [10]. If the TEE is also negative but clinical suspicion remains high, a repeat TEE should be performed for confirmation within five to seven days [8,13]. Delayed diagnosis and treatment of IE can lead to disease progression, resulting in irreparable cardiac injury and significant morbidity and mortality [3].

Once the diagnosis of IE is confirmed, patients should be evaluated by a multidisciplinary team of subspecialists, including cardiology, infectious disease, microbiology, cardiac surgery, and cardiac anesthesiology [1]. The mainstay of IE treatment is early intravenous antibiotic therapy, which may be transitioned to oral therapy, and, when indicated, early surgery to repair or replace affected valves to restore anatomy and cardiac function [14]. Approximately 48.2% of IE cases require surgical intervention, accounting for nearly 25,000 surgical cases annually in the United States [5]. Surgical indications include new-onset heart failure, new-onset valvopathies, uncontrolled or persistent infection, and/or prevention of recurrent septic embolism [3,5]. Surgical considerations include the extent of valvular destruction, infection acuity, hemodynamics, cardiac function, and the patient’s comorbidities [8].

Preoperative considerations

In addition to preoperative echocardiography used for diagnosis, the ESC recommends a preoperative CTA to evaluate coronary obstructions [8]. The AHA also suggests that ECG-gated CT may be useful in evaluating complications of IE, such as abscesses and septic pulmonary infarcts, during preoperative evaluation [3]. The timing and urgency of surgery vary between guidelines, but recent studies suggest that earlier intervention is associated with decreased mortality [15]. In patients with embolic stroke caused by IE, surgery should not be delayed except in the setting of a coma or intracranial hemorrhage [8,16]. In patients with intracranial hemorrhage or coma, surgery should be delayed for at least four weeks due to the survival benefit [8,13].

Intraoperative considerations

Before initiating CPB in valvular surgery, anesthesiologists should perform a preoperative TEE to dynamically assess cardiac and valvular function, identify evidence of infectious spread, evaluate vegetation stability, and guide surgical management [17]. After CPB, anesthesiologists must perform a post-surgical TEE to guide the de-airing of the bypass circuit, assist in CPB weaning, and evaluate the post-surgical repair and function prior to chest wall closure [17]. Antibiotic therapy should be continued intraoperatively, but CPB may decrease plasma antibiotic concentrations. Therefore, consultation with infectious disease specialists or clinical pharmacy should be conducted preoperatively to ensure optimal perioperative outcomes [5]. The heparinization of IE patients undergoing valve surgery with CPB may be complicated by relative heparin resistance caused by IE, and anesthesiologists should collaborate closely with the perfusionist to manage treatment according to institutional algorithms, which may include heparin escalation or antithrombin III administration [18,19]. IE also creates a hypercoagulable state, and in severe cases with sepsis, it may progress into disseminated intravascular coagulation. Intraoperative and postoperative platelet and coagulation studies may be necessary [5]. Anesthesiologists must also be aware of intraoperative hemodynamic instability caused by cardiac dysfunction, acute coronary syndrome, and/or the presence or development of septic shock [5]. Additionally, other end-organ dysfunctions secondary to IE complicating intraoperative management, such as renal, pulmonary, hepatic, and splenic complications, have also been reported in the literature [5].

Postoperative considerations

After valvular surgery, IE patients will be admitted to an ICU for close observation. In addition to post-cardiac surgery care and continued antibiotic therapy, IE patients may require ongoing management of coagulopathy, end-organ dysfunction, septic shock, and refractory heart failure [5].

## Conclusions

A case of IE in an otherwise healthy 58-year-old male, occurring 10 days following a dental procedure, underscores the importance of early recognition and treatment of IE by a multidisciplinary team. IE is a rare, debilitating condition with high in-hospital and 1-year mortality rates. It should be suspected in patients with fever, malaise, and/or new cardiac murmurs in the setting of recent dental interventions, even in the absence of known patient risk factors. Early diagnosis of dental-induced IE can reduce the risk of complications such as heart failure, valvopathy, and septic emboli. However, as seen in the case presented here, nearly half of IE patients will require surgery, regardless of early diagnosis. Surgical management of IE involves several pre-, peri-, and postoperative considerations, among which TEE is a critical tool in the appropriate and effective placement of an artificial valve. Overall, this case serves as a reminder that, while rare, IE carries a high mortality, can occur in patients without known risk factors, and therefore requires a high clinical suspicion to facilitate prompt multidisciplinary treatment.
